# Delivery of Pediatric Cancer Care in Mexico: A National Survey

**DOI:** 10.1200/JGO.17.00238

**Published:** 2018-06-12

**Authors:** Laura Rodriguez-Romo, Alberto Olaya Vargas, Sumit Gupta, Jaime Shalkow-Klincovstein, Lourdes Vega-Vega, Alfonso Reyes-Lopez, Carlo Cicero-Oneto, Juan Mejia-Arangure, Oscar Gonzalez-Ramella, Rafael Pineiro-Retif, Aracely Lopez-Facundo, Maria de los Angeles Del Campo-Martinez, Isidoro Tejocote, Kelly Brennan, Christopher M. Booth

**Affiliations:** **Laura Rodriguez-Romo**, Queen’s University; **Kelly Brennan** and **Christopher M. Booth**, Queen’s University Cancer Research Institute, Kingston; **Sumit Gupta**, The Hospital for Sick Children, Toronto, Ontario, Canada; **Laura Rodriguez** and **Rafael Pineiro-Retif**, Hospital Universitario de la Universidad Autónoma de Nuevo León, Nuevo León; **Alberto Olaya Vargas** and **Jaime Shalkow-Klincovstein**, Instituto Nacional de Pediatria; **Alfonso Reyes-Lopez** and **Carlo Cicero-Oneto**, Hospital Infantil de México; **Juan Mejia-Arangure**, Centro Médico Nacional Siglo XXI; **Maria de los Angeles Del Campo-Martinez**, Centro Médico Nacional La Raza, Mexico City; **Lourdes Vega-Vega**, Hospital Infantil Teletón de Oncología, Querétaro; **Oscar Gonzalez-Ramella**, Hospital Civil de Guadalajara, Guadalajara; **Aracely Lopez-Facundo**, Universidad Autónoma del Estado de México, México; and **Isidoro Tejocote**, Hospital para el Nino del Instituto Materno Infantil, México, México, Toluca, Mexico.

## Abstract

**Purpose:**

Limited data describe the delivery of pediatric cancer care in Mexico. We report a nationwide survey of pediatric cancer units.

**Methods:**

An electronic survey was distributed to 74 pediatric cancer units in Mexico to describe case volumes; organization of care; and availability of medical/surgical specialists, supportive care, complex therapies, and diagnostic services. Centers were classified as low (< 30 new patients/year), medium (30 to 59/year) and high (≥ 60/year).

**Results:**

Sixty-two centers completed the survey (response rate, 84%). The median annual new case volume per center was 50 (interquartile range [IQR], 23 to 81). Thirty-four percent (n = 21), 26% (n = 16), and 40% (n = 25) of units were low-, medium-, and high-volume centers, respectively. Treatment units reported a median of two pediatric oncologists (IQR, 2) and one pediatric hematologist (IQR, 1 to 2). Availability of medical and surgical subspecialists varied by center size, with substantially more specialist support at higher-volume centers (*P* < .01). Multidisciplinary tumor boards are available at 29% (six of 21), 56% (nine of 16), and 76% (19 of 25) of low- to high-volume centers, respectively (*P* = .005). Radiation and palliative care services are available at 42% (n = 26) and 63% (n = 36) of all centers, which did not vary by center volume. Educational support for hospitalized children and school reintegration programs are available at 56% (n = 36) and 58% (n = 36) of centers, respectively. One third (38% [n = 23]) of centers reported that at least one half of patients were lost to follow-up during the transition from pediatric to adult programs.

**Conclusion:**

A large variation exists in annual case volumes across Mexican pediatric cancer centers. Additional efforts to increase access to multidisciplinary, supportive, and palliative care across all pediatric cancer units in Mexico are required.

## INTRODUCTION

Cancer is the second leading cause of mortality among children in Mexico.^1^ Despite being an upper-middle-income country, Mexico has striking levels of income inequality. Among its 123 million inhabitants, 53 million live below the poverty line, and 10 million live in extreme poverty.^2-4^

Pediatric cancer care (PCC) is delivered in diverse settings across Mexico, with substantial variation across centers in available infrastructure and resources. Despite excellent rates of long-term survival achieved for pediatric cancers in high-income countries, outcomes in low- to middle-income and upper-middle-income countries are substantially inferior.^5^ These inferior outcomes relate to multiple factors, including advanced disease at the time of diagnosis, limited access to high-quality cancer care, and high rates of abandonment of therapy. A global study of temporal trends in childhood cancer deaths during 1970 to 2007 reported that the average annual percent change in mortality from all childhood cancers in Mexico was 1%; in developed countries it was −3%.^6^ Although 5-year survival across some pediatric cancers in high-income countries could be as high as 80% to 90%,^7^ data from Mexico report a 50% long-term survival rate.^1^ Another study (2002 to 2013) reported an overall survival of 43%.^8^

In light of these data, the Mexican Association of Pediatric Oncology/Hematology (AMOHP) mandated in 2017 that research capacity be built across Mexico. To date, no national data describe the availability of pediatric cancer services in Mexico. Such baseline data are crucial to set the foundation for strengthening national programs and care delivery systems. Our objective, therefore, was to provide an overview of clinical volumes, infrastructure, and human resource availability for PCC throughout Mexico.

## METHODS

### Setting and Study Population

Mexico has a population of 123 million and comprises 31 states and the capital Mexico City. Health insurance is delivered through five programs: Formally salaried employees and their families (46% of the population) have health insurance through the Instituto Mexicano del Seguro Social, individuals and families with no formal employment (42%) have coverage through popular medical insurance (PMI), federal government workers and families (8%) have health coverage through Instituto de Seguridad Social al Servicio de los Trabajadores del Estado, 2% have coverage through small businesses that provide insurance to their employees; and 3% hold their own private insurance.^9^

The study population included all centers in Mexico that deliver PCC. To identify existing treatment units, centers were identified through three approaches: the AMOHP database, a published report from Mexico’s PMI program, and telephone/e-mail contact of pediatric oncologists in each Mexican state. Seventy-four pediatric cancer units were identified.

### Survey Design and Distribution

An online electronic survey was designed to capture the following information: pediatric oncology case volumes; organization of care; and availability of medical and surgical specialists, supportive care, complex therapies, and diagnostic services. An open-ended question also was included about the challenges faced in delivering PCC. The survey was designed with multidisciplinary input of the study investigators who practice in diverse areas of pediatric oncology. In January 2017, the electronic questionnaire was distributed through Survey Monkey (San Mateo, CA) to a single physician at each of the 74 pediatric cancer units. Follow-up of nonresponses was done through reminder telephone calls and e-mail notices in March and May 2017. The survey was closed on June 6, 2017. The research team reviewed the data, and inconsistencies or ambiguities in survey responses were clarified with direct communication to the reporting center.

### Statistical Analysis

The primary objective was to describe clinical volumes, workforce, and infrastructure of pediatric cancer units in Mexico. Centers were classified as low (< 30 new patients/year), medium (30 to 59 new patients/year), and high (≥ 60 new patients/year) volume. Thirty patients per year was chosen as a benchmark on the basis of the recommended minimum case volume from the European standards of care for children with cancer.^10^ The distinction between medium- and high-volume centers was arbitrarily defined as 60 new patients per year on the basis of the distribution of reported case volumes. All data were initially collected in Survey Monkey and subsequently exported to SAS statistical software (SAS Institute, Cary, NC). Fisher’s exact test was used for differences in proportions among high-, medium-, and low-volume centers because of the presence of cells with fewer than five counts. Comparisons between interval variables were made with the Kruskal-Wallis exact test. Results were considered statistically significant at *P* < .05. All analyses were performed using SAS 9.4. This study was approved by the Research Ethics Board of Queen’s University (Kingston, Ontario, Canada).

## RESULTS

### Survey Response and Center Characteristics

**Fig 1:**
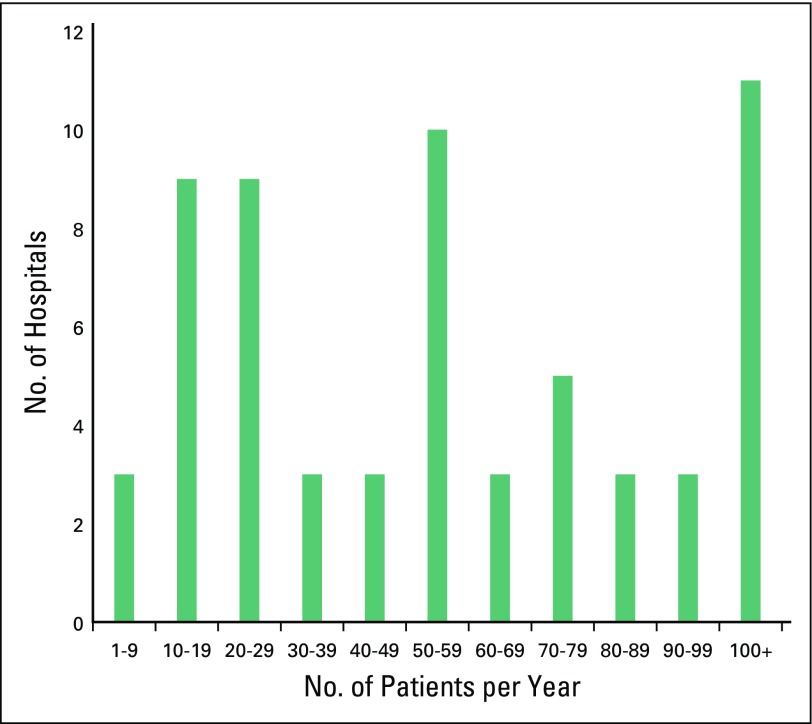
Annual case volume reported by pediatric cancer units (N = 62) in Mexico.

The survey was distributed to 74 pediatric cancer units; 62 units from 29 states completed the survey (84% response rate). The participating units reported seeing approximately 4,225 new consultations per year (median, 50 patients/year, interquartile range [IQR], 23 to 81 patients/year). Considerable variation was found in annual case volumes (range, four to 320 patients/year; Fig 1). Sixty-six percent (n = 41) of the 62 units reported ≥ 30 new consultations per year; these units accounted for 92% (3,873 of 4,225) of all new consultations among the study cohort. All units delivered care for hematologic malignancies, 87% (n = 54) treated solid tumors, and 71% (n = 44) treated CNS tumors. Stem-cell transplantation (SCT) was available at 18% (n = 11) of centers. Formal training programs in pediatric oncology, pediatric hematology, pediatric surgical oncology, and pediatric radiation oncology were offered at seven (11%), seven (11%), three (5%), and five (8%) centers, respectively.

### Providers of PCC

Treatment units reported having a median of two pediatric oncologists (IQR, 1 to 3) and one pediatric hematologist (IQR, 1 to 2); this ranged from a median of one pediatric oncologist and one pediatric hematologist in the low-volume units to a median of three and two providers in the high- and medium-volume units, respectively. Forty-eight centers (77%) reported having pediatric surgical expertise in cancer; in the remaining centers, children are referred elsewhere for surgery. Only 25 centers (40%) had a radiation oncologist with specific pediatric expertise. Palliative medicine physicians were available at 48% (n = 30) of centers. Higher-volume centers had a considerably greater number of pediatric oncologists/hematologists and a greater number of medical and surgical subspecialists. Multidisciplinary tumor boards (MDTBs) were available in 55% (n = 34) of all units. MDTBs were available in 29% (six of 21), 56% (nine of 16), and 76% (19 of 25) of low-, medium-, and high-volume units, respectively (*P* = .005; Table1).

**Table 1:**
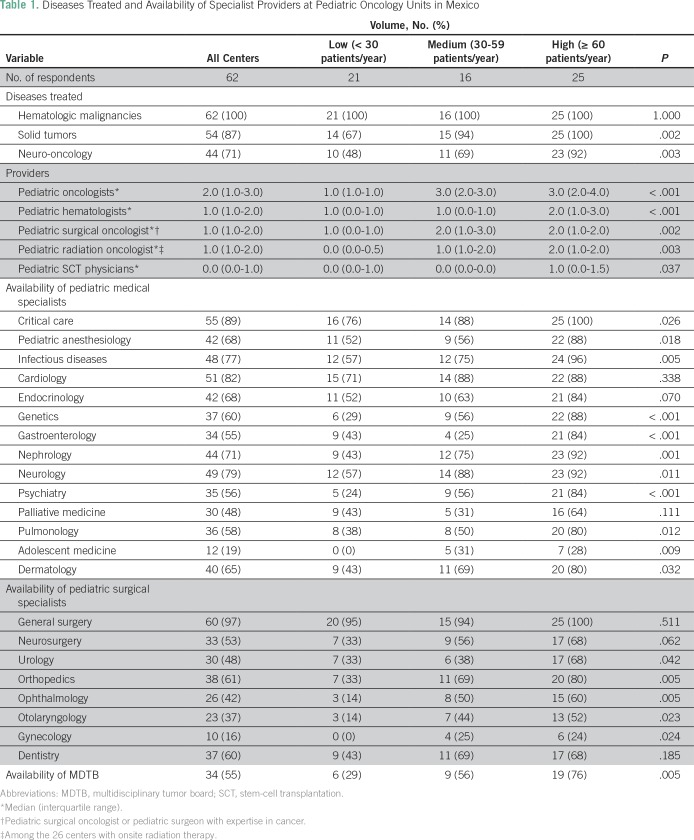
Diseases Treated and Availability of Specialist Providers at Pediatric Oncology Units in Mexico

### Availability of Clinical Services

The median number of dedicated inpatient beds per center was 10; this number varied across centers, with a median of seven (IQR, 5 to 8), 10 (IQR, 8 to 14), and 22 (IQR, 12 to 30) beds (*P* < .001) at low-, medium-, and high-volume centers, respectively (Table 2). The median number of ward nurses per inpatient bed was four (IQR, 4 to 5). Seventy-four percent (n = 46) of the 62 centers have immediate access to critical care services, 16% (n = 10) reported usually having immediate access, and 10% (n = 6) do not have immediate access. Eleven centers (18%) offered SCT. Forty-two percent (n = 26) of units have radiotherapy available on site, 73% (19 of 26) of which have linear accelerators. Palliative care clinics are available at 63% (n = 39) of units, and this did not vary by center size (*P* = .799). Palliative care physicians are available at 48% (n = 30) of units, and there was a trend toward greater availability at higher-volume centers (*P* = .111).

**Table 2:**
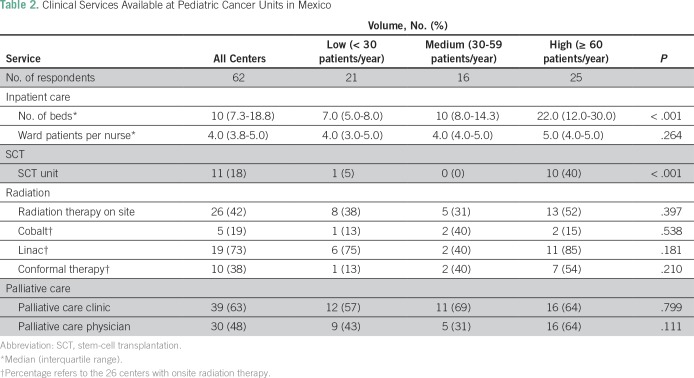
Clinical Services Available at Pediatric Cancer Units in Mexico

### Availability of Diagnostic Services

Seventy-one percent (n = 44) of the 62 centers have board-certified radiologists. Radiography and ultrasound were available in 100% of centers; 94% (n = 58) have computed tomography, 58% (n = 36) have magnetic resonance imaging, 21% (n = 13) have positron emission tomography, and 34% (n = 21) have radionuclide imaging.

Seventy-one percent (n = 44) of the 62 centers reported having board-certified pathologists with expertise in pediatric cancer. Available diagnostic services include flow cytometry in 76% (n = 47), cytogenetics in 60% (n = 37), fluorescent in situ hybridization in 47% (n = 29), reverse transcription polymerase chain reaction in 50% (n = 31), and immunohistochemistry in 74% (n = 46).

All centers reported having general clinical laboratory services. Blood banks capable of providing a full range of products, including irradiated and leukodepleted blood components, were available on site at 76% (n = 47) of the 62 centers; other centers relied on external blood bank units. In 35% (n = 22) of units, chemotherapy drugs were mixed by pharmacists; in the remaining centers, the mixing was done by the nurses. Only 32% (n = 20) of centers had the facilities to monitor antineoplastic drug concentrations (Table 3).

**Table 3:**
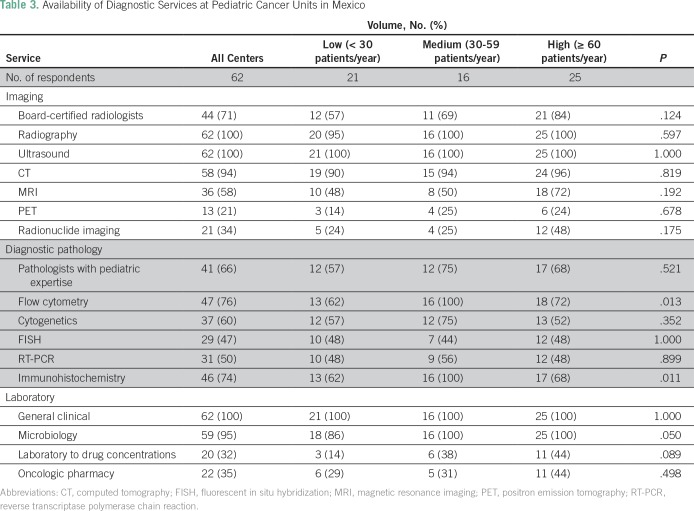
Availability of Diagnostic Services at Pediatric Cancer Units in Mexico

### Follow-Up, Survivorship, and Supportive Care Services

The upper age limit treated in pediatric treatment units was 17 years in 45 centers (73%), 16 years in five centers (8%), 15 years in nine centers (15%), and 14 years in three centers (5%). Fifty-five percent (n = 34) of the 62 centers transition patients to adult clinics located in the same hospital; 42% (n = 26) transfer patients to adult clinics at another hospital. One third (31% [n = 19]) of centers reported receiving updates on transferred patients from the adult clinics. Thirty-eight percent (n = 23) reported a substantial number (≥ 50%) of transferred patients being lost to follow-up. Late-effects clinics were only available at 13% (n = 8) of units. Social workers were available at 52% (11 of 21), 94% (15 of 16), and 92% (23 of 25) of low-, medium-, and high-volume centers, respectively (*P* = .002; Table 4).

**Table 4:**
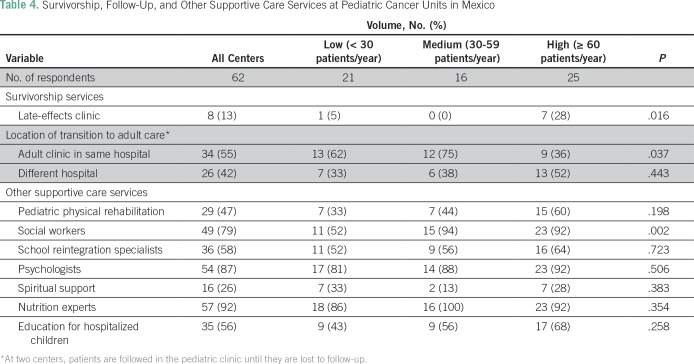
Survivorship, Follow-Up, and Other Supportive Care Services at Pediatric Cancer Units in Mexico

### Self-Reported Challenges in Delivery of Care

Commonly reported challenges among the 62 units were barriers within hospital administration (44% [n = 27]), access to diagnostic services (29% [n = 18]), patient-level barriers (21% [n = 13]), availability of hematology/oncology services (21% [n = 13]), lack of a treatment/research network (21% [n = 13), lack of multidisciplinary support (19% [n = 12]), access to treatment (19% [n = 12]), and limited infrastructure (10% [n = 6]; Table 5).

**Table 5:**
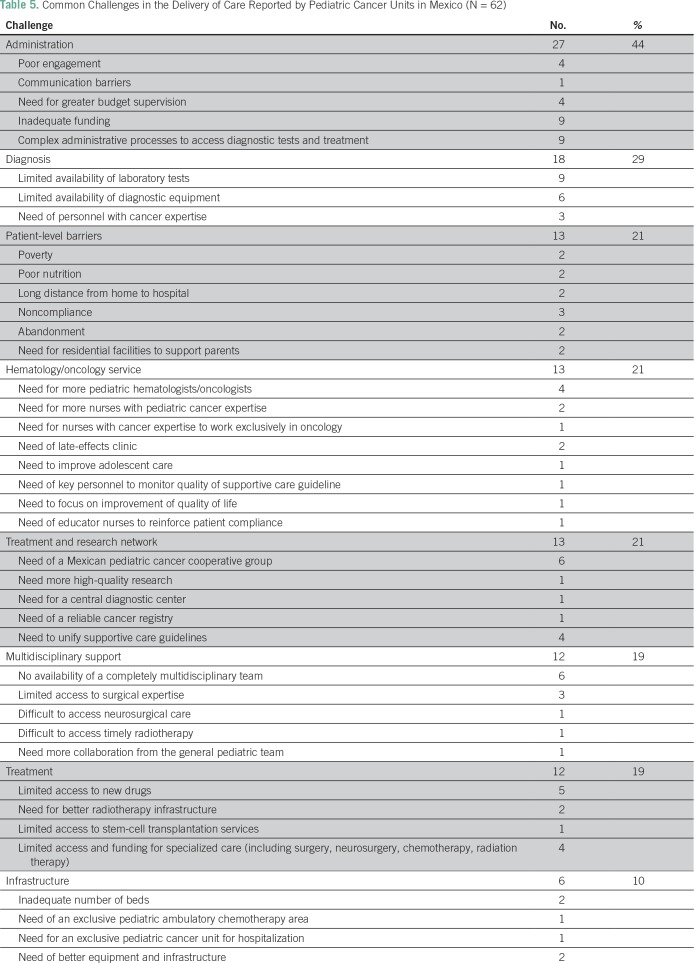
Common Challenges in the Delivery of Care Reported by Pediatric Cancer Units in Mexico (N = 62)

## DISCUSSION

We describe the organization of PCC in Mexico, and several important findings have emerged. First, considerable variation exists in annual case volumes, with one third of units treating < 30 patients per year. Second, availability of specialist providers, MDTBs, and core diagnostic services is greater at medium- and high-volume centers compared with low-volume centers. Third, radiation services and palliative care clinics are only available at 42% and 63% of all centers, respectively. Fourth, one third of centers reported that at least one half of patients are lost to follow-up during the transition from pediatric to adult programs. Finally, commonly reported barriers to delivery of care relate to administration, patient socioeconomics, and limited access to specialized diagnostic and therapeutic services.

These data may be useful for future planning exercises. With an 84% response rate, the study findings likely are generalizable to the overall Mexican childhood cancer care system. The 62 centers included in this study reported a total annual case volume of 4,225 patients. Application of the median case volume (50 per year) to the remaining 12 centers suggests approximately 4,825 new cases of pediatric cancer per year in Mexico. With a national population of approximately 39.2 million children (< 18 years of age), these figures generate an annual incidence of childhood cancer of approximately 123 per million, which is consistent with the estimated incidence generated by Fajardo-Gutiérrez et al^8^ and with the official numbers of the PMI reported by Rivera-Luna et al.^11^ Recently published data have shown that annual incidence rates among 0- to 14-year olds may vary from < 50 per million in sub-Saharan Africa to 155 to 175 per million in Western Europe.^12^ Although unable to account for biologic differences in incidence, the current data suggest that underdiagnosis is not as significant a problem in Mexico as it is in other low- and middle-income countries.

Center and oncologist/hematologist annual case volumes are comparable with data reported in a survey of European centers.^10^ Data from 321 pediatric cancer units across 35 countries showed a mean center and oncologist/hematologist annual case volume of 54 and 19 new patients, respectively; comparative numbers from the current study are 68 and 17. These figures are higher than the recommended annual case volume of 15 new patients per oncologist proposed by the Council of Canadian Pediatric Hematology/Oncology and Transplantation Directors.^13^ The ratio of ward nurses to pediatric cancer inpatients (one to four) is consistent with the recommended benchmark (one to five) proposed by the International Society for Pediatric Oncology.^14^ However, the current data do not offer insight into the expertise and/or training of nurses who staff pediatric oncology wards in Mexico.

The European Society for Pediatric Oncology proposed a minimum annual case volume of 30 new patients per center.^10^ One third of centers in this study do not meet this threshold. However, our study provides some reassurance because > 90% of all children were treated at centers that exceeded this benchmark. The results demonstrate the greater availability of medical and surgical specialty care at larger centers but do not provide insight into whether a volume-outcomes relationship exists in PCC. Data in this field are limited but suggest that outcomes of complex pediatric surgical procedures are superior at higher-volume centers.^15,16^ In addition, key supportive care services, such as social work, were more commonly found in higher-volume centers than in lower-volume centers (92% *v* 52%; *P* = .002). Given the known role of psychosocial care in decreasing rates of treatment abandonment,^17,18^ rates of abandonment may well be higher at lower-volume centers, although this hypothesis remains unproven. Additional work is needed within the Mexican context to determine whether centralization of care would lead to improved outcomes; the potential downside of this process would be to decrease access and increase the proportion of patients who do not seek care in a timely manner. The establishment of satellite centers associated with primary cancer centers, as implemented in other jurisdictions,^19^ may represent a balance between these two priorities but requires coordinated regional and national networks of care. Most smaller centers in Mexico deliver treatment to patients with leukemia and solid tumors. However, patients with more complex needs (ie, SCT, radiation, complex surgery) will be referred to larger centers.

Forty-five percent of centers did not have an MDTB, with availability ranging from 29% in low-volume centers to 76% in high-volume centers. MDTBs are known to improve decision making and the quality of care delivered to children with cancer.^20^ A current AMOHP initiative is to build formal relationships between smaller nonacademic centers and larger academic units to facilitate joint MDTBs and other models of collaborative care.

Palliative care services are available at 63% of centers, but not all have palliative medicine physicians. A growing body of literature supports the role of palliative care in improving patient and caregiver outcomes, including quality of life and even survival.^21^ Moreover, the principles of palliative care can be applied successfully and can be cost-effective, even in resource-limited settings.^22^ In 2014, the General Health Council of Mexico declared an obligation to provide palliative care services to patients in need.^23^ Improvement of access to palliative care will continue to be a focus of AMOHP. Despite the high proportion of centers that lack access to onsite palliative care and radiation oncology services, these were not commonly reported as major barriers to care (Table 5); thus, centers without these critical services possibly have relatively good access to palliative care at nearby institutions.

A substantial proportion of centers lack educational supports for patients. Continued education and school reintegration, therefore, are areas that require improvement because the health system supports long-term development and success of children with a history of cancer. Less than half of the centers had access to pediatric physical rehabilitation; this service requires additional expansion to improve health-related quality of life in both physical and psychological dimensions.^24^

The Children’s Oncology Group has developed long-term follow-up guidelines for survivors of childhood, adolescent, and young adult cancers.^25^ Young adult survivors of childhood and adolescent cancer are a growing population, and many remain at lifelong risk of potentially serious complications of their cancer therapy. Management of this unique group requires a broad-based interdisciplinary clinical team. Despite this, data from the current study suggest that a substantial proportion of adolescents are lost to follow-up in the transition from pediatric to adult follow-up programs and may be partially explained by insurance coverage within many health programs in Mexico not extending beyond 17 years. The extent to which these economic barriers negatively affect the care of childhood cancer survivors in Mexico requires additional study. Pediatric cancer centers in Mexico should develop formal programs for young adult survivors in partnership with neighboring adult institutions. The high proportion of patients who are lost to follow-up at the time of transition to adult centers is concerning. The current survey results do not offer insight into the root causes of this problem. One of the goals of this study was to generate preliminary data that will allow AMOHP to identify problems and undertake more-detailed analyses so that strategies can be implemented to improve current models of care, which may involve the creation of late-effects clinics at all pediatric units as well as more integrated electronic records that can follow the patient from one center to the next. AMOHP will consider programs such as the Survivor Passport^26^ initiative in Europe to close these gaps in care.

The study results should be considered in light of methodological limitations. First, the most notable limitation is the self-reported nature of the data, including case volumes and available services. Our approach also may have led to some double counting of patients who were referred from one center to another. Second, not all pediatric units in Mexico responded to the survey. However, because our response rate was excellent, the results likely are generalizable across Mexico. None of the nonresponder centers were academic units, and all are small- and medium-sized units. Third, the survey was sent to only a single individual at each institution; if we had included more than one individual at the center, we may have had an improved response rate. Finally, the reported availability of services from our survey does not offer insight into the quality of those services or their relative accessibility and timeliness. Future work should explore in more detail the commonly reported barriers to high-quality care at the patient, provider, and system level. Disease-specific and more granular patient-level and treatment data would enable a more complete study of patterns of care and outcomes achieved in Mexico.

This study provides important insights into the delivery of PCC in Mexico. Case volumes vary substantially across centers as does the availability of specialized services. Additional capacity in supportive and palliative care is needed. The data provide a starting point for future quality-of-care initiatives to improve outcomes of children with cancer in Mexico.
